# P-TarPmiR accurately predicts plant-specific miRNA targets

**DOI:** 10.1038/s41598-022-27283-8

**Published:** 2023-01-06

**Authors:** Victoria Ajila, Laura Colley, Dave T. Ste-Croix, Nour Nissan, Ashkan Golshani, Elroy R. Cober, Benjamin Mimee, Bahram Samanfar, James R. Green

**Affiliations:** 1 Department of Systems and Computer Engineering, Carleton University, Ottawa, K1S 5B6 Canada; 2 Saint-Jean-sur-Richelieu Research and Development Center, Agriculture and Agri-Food Canada, Saint-Jean-sur-Richelieu, J3B 7B5 Canada; 3 Ottawa Research and Development Center, Agriculture and Agri-Food Canada, Ottawa, K1A 0C6 Canada; 4 Department of Biology, Carleton University, Ottawa, K1S 5B6 Canada

**Keywords:** Gene expression, Machine learning, Computational biology and bioinformatics, Software

## Abstract

microRNAs (miRNAs) are small non-coding ribonucleic acids that post-transcriptionally regulate gene expression through the targeting of messenger RNA (mRNAs). Most miRNA target predictors have focused on animal species and prediction performance drops substantially when applied to plant species. Several rule-based miRNA target predictors have been developed in plant species, but they often fail to discover new miRNA targets with non-canonical miRNA–mRNA binding. Here, the recently published TarDB database of plant miRNA–mRNA data is leveraged to retrain the TarPmiR miRNA target predictor for application on plant species. Rigorous experiment design across four plant test species demonstrates that animal-trained predictors fail to sustain performance on plant species, and that the use of plant-specific training data improves accuracy depending on the quantity of plant training data used. Surprisingly, our results indicate that the complete exclusion of animal training data leads to the most accurate plant-specific miRNA target predictor indicating that animal-based data may detract from miRNA target prediction in plants. Our final plant-specific miRNA prediction method, dubbed P-TarPmiR, is freely available for use at http://ptarpmir.cu-bic.ca. The final P-TarPmiR method is used to predict targets for all miRNA within the soybean genome. Those ranked predictions, together with GO term enrichment, are shared with the research community.

## Introduction

microRNAs (miRNAs) are a class of short non-coding ribonucleic acids (RNAs) 20 to 24 nucleotides in length that achieve post-transcriptional gene expression regulation^1–3^. miRNAs are created through a multi-step process that includes the formation of pre-miRNA (precursor miRNA) sequences before the final processing step creating mature miRNA^2,4^. miRNAs regulate gene expression through the ribonucleoprotein complex (RISC)^3,4^. The miRNA-RISC complex binds to its target messenger RNA (mRNA) inducing its silencing or degradation through translational repression which can be coupled with mRNA decay and RISC-catalyzed endonucleolytic mRNA cleavage^2,4,5^.

Biochemical assays including western blots, microarrays, next-generation sequencing, and quantitative polymerase chain reaction have been used to successfully determine miRNA targets at the gene level^6^. However, these techniques are unable to determine the exact binding site of miRNA within the mRNA^7^. Other experimental techniques, such as HITS-CLIP and PAR-CLIP can identify specific target sequences^8^. Cross-linking ligation and sequencing hybrids (CLASH) is a high throughput experimental approach that simultaneously identifies miRNA target sequences and the corresponding miRNA^9^. Cross-linking and immunoprecipitation (CLIP) is a high throughput profiling data technique that identifies transcript targets associated with functional RISC complexes; however, the discovery of miRNA target sites does not guarantee functional target suppression^10^. High-quality high-throughput data from wet-lab experimentation are essential for the improvement of computational miRNA target prediction methods^10^. Experimental observations have led to widely used miRNA target prediction rules that describe important features in a probable target^1^.

Given the complexity and cost of experimental techniques, several computational methods for predicting miRNA targets have been developed. The computational methods can be organized into ab initio, machine learning, and hybrid methods^1^. The ab initio methods are designed to apply experimentally-derived rules that examine sequence complementarity in the seed region and other features including the accessibility of the target site, AU content, folding energy, conservation, perfect pairing of the miRNA 5$^{\prime }$ end, and low GC content in the target site^1,8^. TargetScan and miRanda are the two most widely used ab initio miRNA target prediction tools^8^. MiRanda^9^ uses an estimated sequence complementarity score, sequence conservation, and free energy values to predict target sites^1,8^. TargetScan^10^ looks for perfect seed matches to form a candidate target list then uses site-type, local AU enrichment, and other features to calculate a target score for each candidate^1^.

In both plants and animals, miRNAs regulate gene expression by controlling regulatory genes; however, there are many differences between the two kingdoms concerning miRNA biogenesis, miRNA–mRNA binding, and method of mRNA control^11^. Plant miRNA typically require much higher sequence complementarity than animals in the seed region^11^. Additionally, homology-based searches of similar miRNA–mRNA relationships in similar species are much more successful in plants than in animals^11^. Notably, the location of miRNA binding sites on mRNA is different between plants and animals. Animal miRNA bind in the 3$^{\prime }$ UTR region of the mRNA and can exhibit multiplicity, where one mRNA can have many miRNA binding sites and one miRNA can target multiple mRNAs^4,11^. Whereas a plant miRNA binds to the target gene’s open reading frame and there is typically only one binding site per mRNA^2,4,11^.

Plant-specific ab initio miRNA target predictors have also been developed. psRNATarget^12^ is a method that uses the RNAup algorithm^13^ and a modified Smith–Waterman algorithm to find high-confidence miRNA targets^14^. Other plant-specific algorithms like Targetfinder^15^, TAPIR^16^, and Target-align^17^ use the FASTA or Smith–Waterman algorithm accompanied with scoring methods to discover high confidence interactions^14^. The combination of psRNATarget and Targetfinder has resulted in improved performance^14^. Plant miRNA targeting was initially thought to be simple since high seed region complementarity is a requirement for effective gene silencing; however, deviations from the experimentally defined rules have been reported^18^.

Traditionally, machine learning algorithms apply a classifier trained on features extracted from experimentally verified data to filter candidate predictions arising from ab initio algorithms^1^. Some of these machine learning methods include RFMirTarget^19^ (a random forest classifier), MultiMiTar^20^ (an SVM classifier), NBmiRTar^21^ (a hybrid Naïve Bayes classifier), MiRAW^22^ (deep learning), DeepMirTar^6^ (stacked denoising autoencoders), and MiRDTL^23^ (convolutional neural networks).

TarPmiR is a random-forest-based approach that integrates six conventional features with seven new features to predict miRNA target sites^8^. TarPmiR first extracts candidate target sites using miRanda^9^ or other ab initio methods, then extracts 13 features for prediction^21,24^. These features include folding energy, seed match accessibility, AU content, stem conservation, flanking conservation, m/e motif, the total number of paired positions, the length of the target mRNA region, the length of the largest consecutive pairings, the position of the largest consecutive pairings relative to the 5$^{\prime }$ end of miRNA, the number of paired positions at the miRNA 3$^{\prime }$ end, the difference between the number of paired positions in the seed region and that in the miRNA 3$^{\prime }$ end^8^. The algorithm was developed through a thorough validation and feature selection process which determined the best machine learning model and most important features^8^. TarPmiR performed better than both TargetScan and miRanda, two of the most commonly used miRNA target prediction tools when tested on two datasets from the human HEK293 cell line, a mouse dataset, and a general human dataset^8^. Their results also suggest that miRanda and TargetScan do not accurately predict non-seed-matching binding sites^9^.

miRNA–mRNA interactions, predicted by the methods mentioned above and others, are aggregated in several databases, including EIMMo, DIANA-microT, Microcosm, Microrna.org, MirDB, PITA, TargetScan, miRWalk-predictive, and TargetSpy^1^ all of which contain stricly animal interactions. Experimentally validated miRNA–mRNA pairs can be found in repositories such as miRWalk^25^, miRecords^26^, TarBase^27^, miRTarBase^28^, and starBase^29^. Of these databases, only TarBase and miRTarbase list plant interactions in addition to animal interactions.

Most machine learning miRNA–mRNA target prediction algorithms are based on training data derived from organisms in the *Animalia* kingdom. However, a small number of methods are amenable to fine-tuning or retraining using data from species closely related to the target species. Specifically, the TarPmiR method can be adapted to extract 11 of the 13 required features from custom datasets of miRNA–mRNA interactions with known binding sites. However, the retraining of machine learning models requires a significant amount of miRNA–mRNA interaction data, which has thus far been limited for most plant species. TarDB^30^ is a newly released database containing tens of thousands of high-confidence plant miRNA–mRNA interactions. Although the database was originally created for biologists to use for manual homology-based target analysis, we here demonstrate that it can be used to retrain a machine learning method to create a highly accurate plant-specific miRNA target prediction pipeline.

In this study, TarPmiR, a state-of-the-art animal-based miRNA target predictor is modified and retrained for use on plants. TarDB, a new plant miRNA–mRNA database is used for the first time to train a miRNA target predictor. Negative miRNA–mRNA interaction examples are derived from positive miRNA–mRNA interaction examples to form comprehensive training and evaluation datasets. Rigorous experiment design is used to demonstrate that the inclusion of plant interaction data, and the complete exclusion of animal interaction data, significantly improves miRNA target prediction performance across four plant species. Our experiments also determine that a large amount of plant interaction data is required to significantly improve prediction performance. Our final method, dubbed P-TarPmiR, is available for use at http://ptarpmir.cu-bic.ca. The final predictor is applied to all miRNA in the soybean genome and ranked targets are shared with the research community at https://doi.org/10.5683/SP3/LOD4E3. GO term enrichment analysis is completed among all predicted gene targets for each miRNA, in an effort to elucidate the function of each miRNA.

## Results

TarPmiR is a miRNA target predictor traditionally trained on the Human CLASH dataset. In this study, four classifiers with the same model architecture as TarPmiR but different training data were trained and tested in four different experiments. The experiments were designed to ascertain the effect of including plant interaction data in the training dataset on the plant miRNA target prediction performance of the classifier. Four classifiers of varying proportions of plant interaction data were trained, including an animal-based classifier, a multi-kingdom classifier with minimal plant data, a multi-kingdom classifier significantly augmented with plant data, and a strictly plant-based classifier. Each experiment consisted of applying a classifier to the four different test sets. These test sets consisted of positive and negative miRNA–mRNA interaction examples from one of the four most represented organisms in the TarDB dataset: *Glycine max* (*gma*), *Oryza sativa* (*osa*), *Populus trichocarpa* (*ptc*), and *Brachypodium distachyon* (*bdi*).

Rigorous experiments were designed to ensure low sequence homology between the training data of the training sets and the test sets. This was used to simulate the case where an unannotated species is analyzed. For each plant species, it was ensured that none of the training data was similar to any known interaction in the test data. All miRNA included in the TarDB dataset were first clustered by CD-HIT using a sequence identity threshold of 70%.

The miRNA and mRNA sequence data of the interactions in the *H. sapiens*, *A. thaliana*, and TarDB datasets were retrieved. Some mRNA listed in the datasets could not be retrieved due to their removal from current genome annotations. The negative target sites were extracted from the retrievable mRNA and the features were extracted from all positive and negative interactions (see “Methods” section for further detail). The composition of the *H. sapiens* dataset, the *A. thaliana* dataset, and the TarDB dataset are listed in Table 1.

**Table 1:** The composition of the miRNA–mRNA interaction datasets used to develop model training sets, reporting the number of interacting (positive) and non-interacting (negative) miRNA:mRNA sequence pairs.

Training set	*H. sapiens* dataset	*A. thaliana* dataset	TarDB dataset
Positive	17,187	68	40,483
Negative	12,198	53	36,940

Four classifiers with the same model architecture as TarPmiR but differing training sets were trained and tested in four plant species. As described in the “Methods” section, training data from each experiment excluded miRNA–mRNA interactions similar to miRNA from the test species. In each experiment, the test dataset contained representative miRNA from one of the four target organisms (*gma*, *osa*, *ptc*, and *bdi*).

In the first experiment, the “Human” classifier was trained on the “Human” training set comprising only *H. sapiens* data. The “Human+ath” classifier was trained on the “Human+ath” dataset consisting of the *H. sapiens* and *A. thaliana* datasets. The four “Human+Plant” classifiers were trained on the H. sapiens dataset augmented with TarDB plant training data for each plant species. Finally, the “Plant” classifiers were trained using only plant training data from TarDB. The composition of the training sets and test sets for the four test species in the four experiments are listed in Table 2. Notably, there is a drastic decrease between the size of the TarDB dataset shown in Table 1 and the training datasets available for the “Plant” classifiers. TarDB contains many cross-species conserved miRNA targets, which results in a large reduction of the training dataset when all interactions involving a miRNA with 70% similarity or larger are removed^30^

**Table 2:** The composition of the training sets and test sets used to train four classifiers (Human, Human+ath, Human+Plant, and Plant) for application on four test sets (*Glycine max*, *Oryza sativa*, *Populus trichocarpa*, and *Brachypodium distachyon*).

Training set	Human training set	Human+*ath* training dataset	Human+Plant training set				Plant training set				Test set			
			*gma*	*bdi*	*osa*	*ptc*	*gma*	*bdi*	*osa*	*ptc*	*gma*	*bdi*	*osa*	*ptc*
Positive	17,187	17,255 (68 from *ath*)	22,078 (4891 from TarDB)	25,285 (8098 from TarDB)	23,123 (5936 from TarDB)	23,098 (5911 from TarDB)	4891	8098	5936	5911	3939	1664	1237	2761
Negative	12,198	12,251 (53 from *ath*)	16,527 (4329 from TarDB)	19,572 (7374 from TarDB)	17,494 (5296 from TarDB)	17,638 (5440 from TarDB)	4329	7374	5296	5440	3635	1616	1174	2540

**Figure 1:**
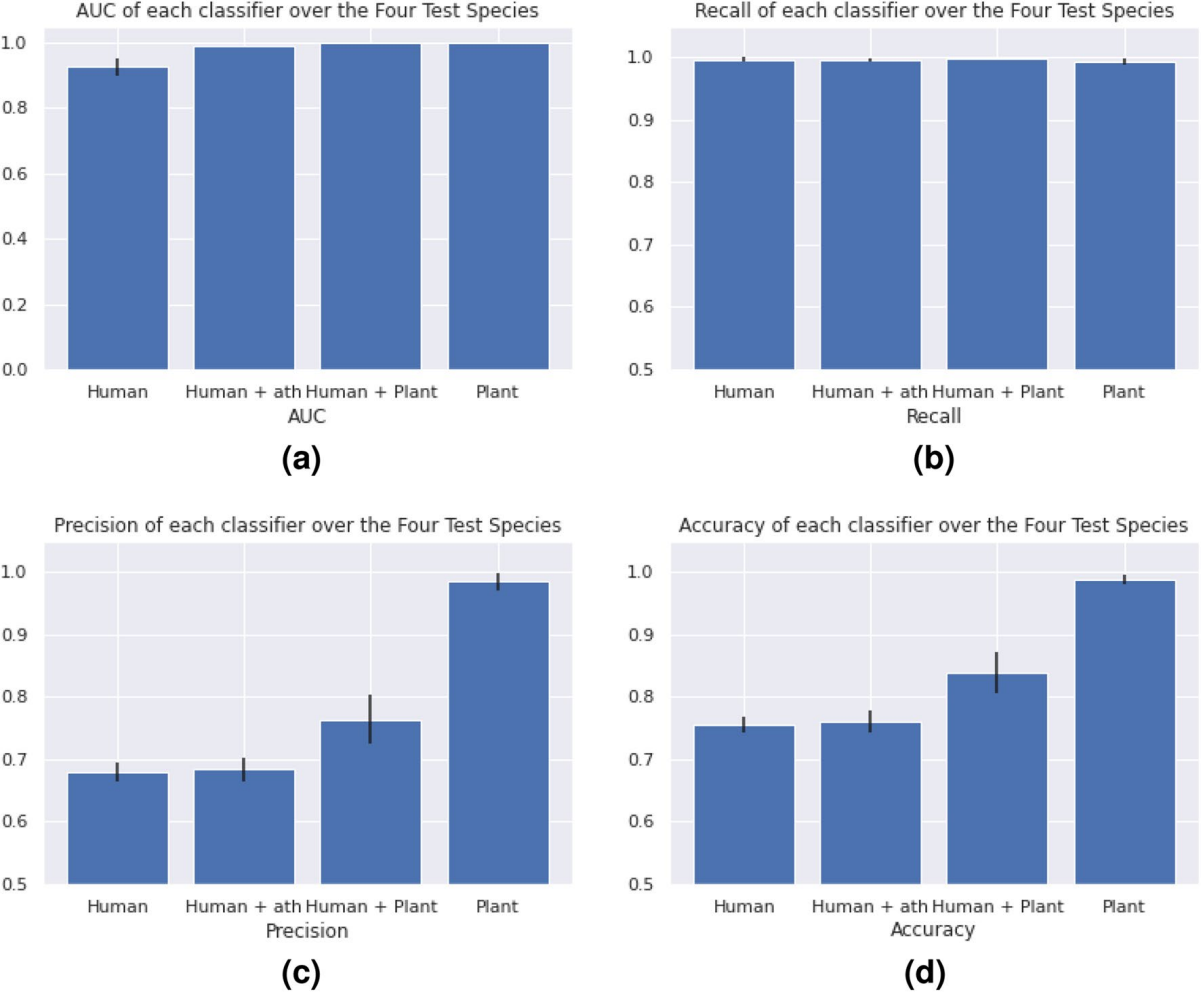
The average value and standard deviation of the performance metrics of each classifier (Human, Human+ath, Human+Plant, and Plant) over the four test species (*Glycine max*, *Oryza sativa*, *Populus trichocarpa*, and *Brachypodium distachyon*). The performance metrics included are area under the Precision–Recall curve (AUC), recall (Re), Precision (Pr), and accuracy (ACC).

Table 3 summarizes the performance of the four classifiers on the test sets in terms of area under the Precision–Recall curve (AUC), recall (Re), Precision (Pr), and accuracy (ACC), where the latter three metrics were evaluated at a confidence threshold of 0.5. Classifiers that included a large number of plant interactions (i.e., “Human+Plant” and “Plant”) performed the best in terms of AUC, recall, precision, and accuracy. Fig. 1 compares the average of each performance metric of the four classifiers.

**Table 3:** Performance of the four (Human, Human+ath, Human+Plant, and Plant) classifiers in each of the four test plant species (*Glycine max*, *Oryza sativa*, *Populus trichocarpa*, and *Brachypodium distachyon*).

Org./exp.	Human				Human+ath				Human+Plant				Plant only			
	AUC	Re	Pr	Acc	AUC	Re	Pr	Acc	AUC	Re	Pr	Acc	AUC	Re	Pr	Acc
*gma*	0.934	0.992	0.697	0.772	0.937	0.991	0.695	0.769	0.997	0.998	0.788	0.859	1.000	0.986	0.995	0.990
*bdi*	0.934	0.996	0.663	0.741	0.939	0.997	0.660	0.738	0.998	0.999	0.744	0.825	0.999	0.996	0.993	0.994
*osa*	0.932	1.000	0.677	0.755	0.928	0.997	0.679	0.757	0.998	0.999	0.720	0.800	0.999	0.997	0.963	0.978
*ptc*	0.959	0.999	0.682	0.757	0.964	0.998	0.703	0.779	0.998	1.000	0.804	0.873	0.996	0.996	0.987	0.991
Average	0.939	0.996	0.680	0.756	0.942	0.996	0.684	0.761	0.998	0.999	0.764	0.839	0.999	0.993	0.984	0.988

**Figure 2:**
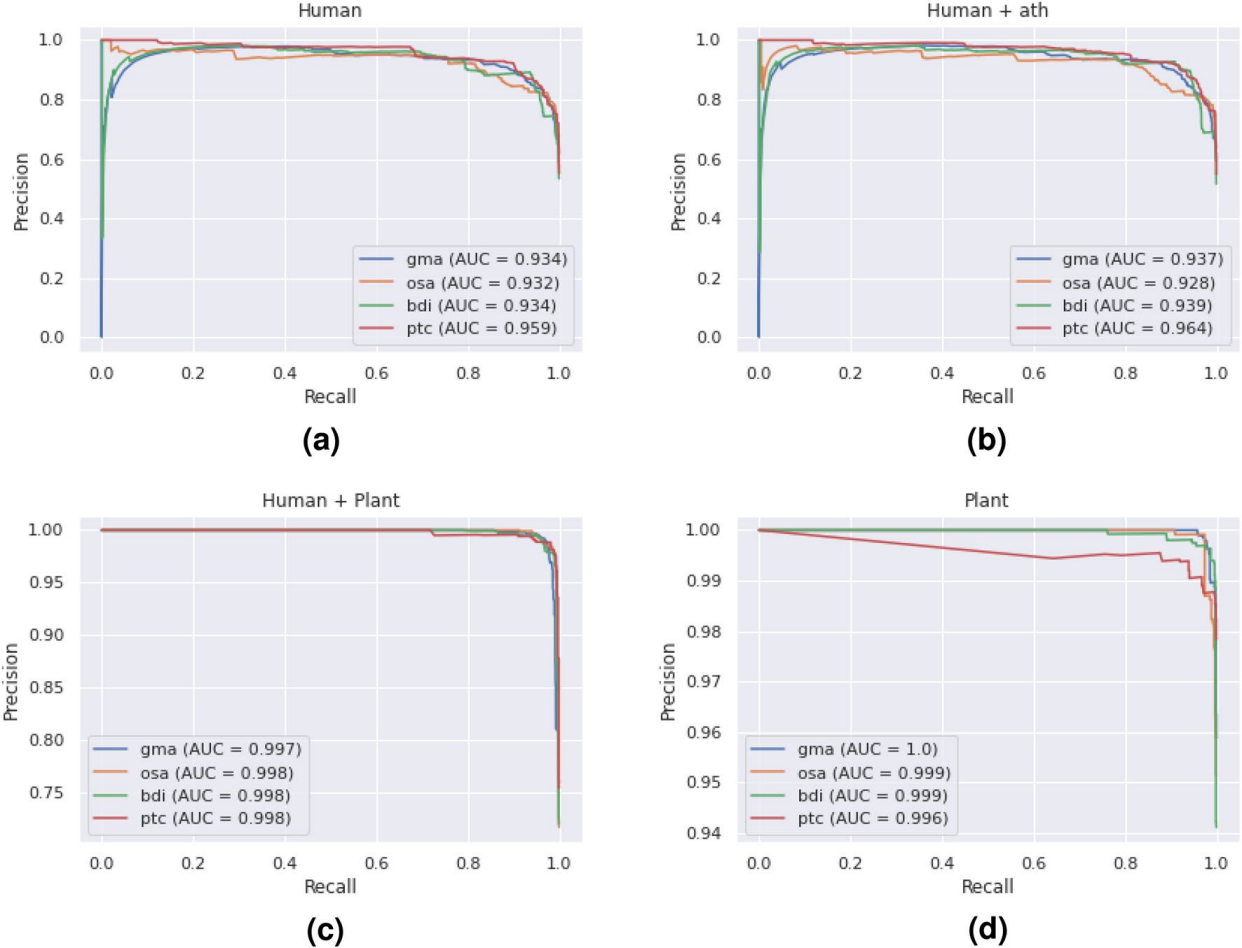
The Precision–Recall curves of each of the four classifiers (Human, Human+ath, Human+Plant, and Plant) for the four plant test species (*Glycine max*, *Oryza sativa*, *Populus trichocarpa*, and *Brachypodium distachyon*).

**Figure 3:**
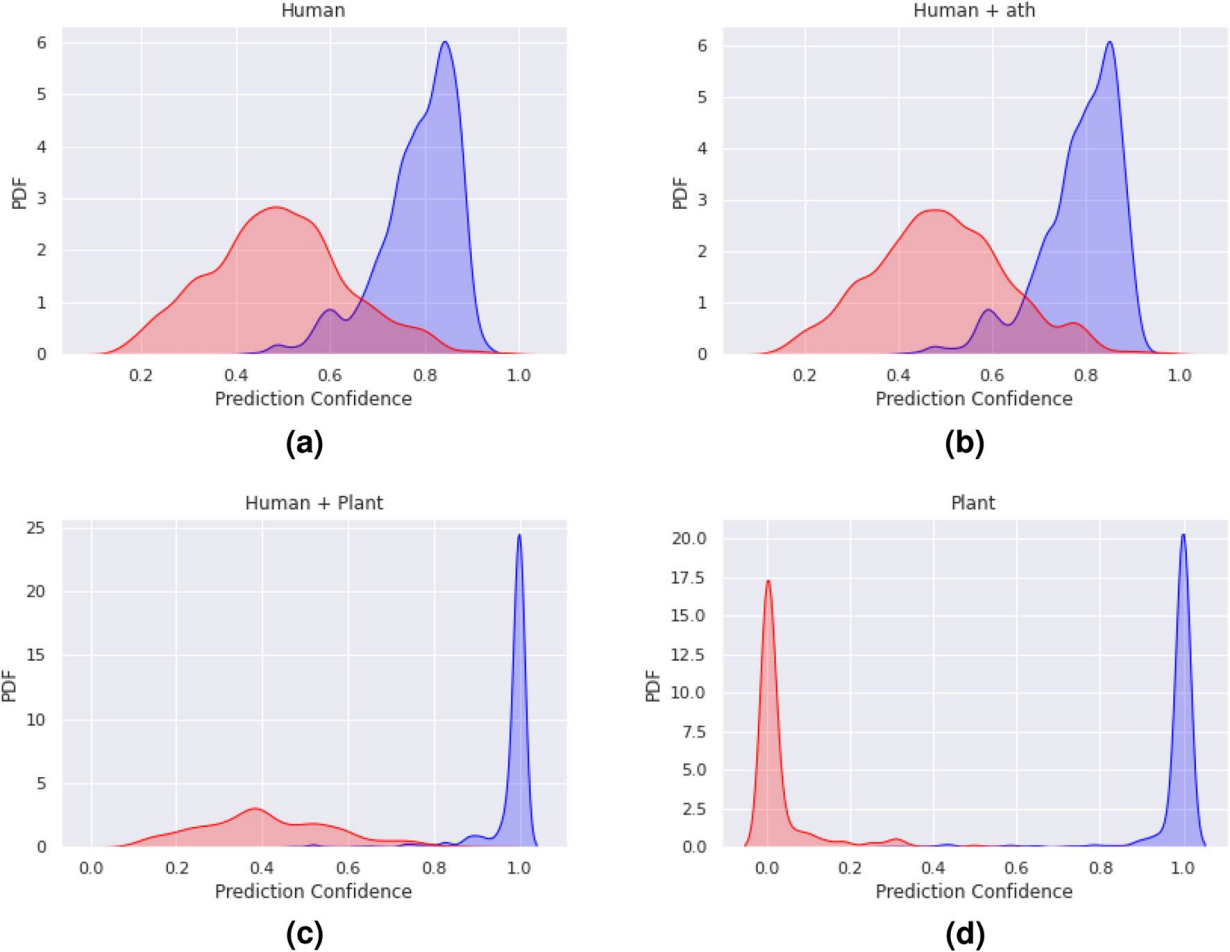
Density plots of the prediction scores of the four classifiers (Human, Human+ath, Human+Plant and Plant) on the *gma* test set. Here, prediction scores for negative test samples are shown in red, while positive test sample scores are shown in blue. A stronger classifier will lead to greater discriminability between the scores generated from positive and negative test miRNA:mRNA pairs.

Figure 2 contains the Precision–Recall curves of the four classifiers over all experiments. As more plant interaction data are included in the training sets of the classifiers, the AUC of the Precision–Recall curve increases. Notably, the AUC on the *ptc* test set of all the experiments was greater than other test sets (except the plant-only case). ANOVA tests ($\hbox {p} < 0.05$) found that the AUC, precision, and accuracy of the classifier results listed in Table 2 were statistically significantly different. Post hoc paired t-tests showed that the performance of the “Human+Plant” and “Plant” classifiers was significantly different from the “Human” and “Human+ath” classifiers. However, the performance of the “Human” and “Human+ath” classifiers was not significantly different from each other. Conversely, the “Human+Plant” and “Plant” classifiers were significantly different from each other, except for the AUC and recall performance metrics. Figure 3. displays the density plots for the classifiers on the *gma* test set. Here, a wider separation between negative and positive scores is desirable. In line with the results in Table 3 and Fig. 2, as more plant interaction data are included in the training sets, the separation between the positive and negative test data increases.

### Web server

**Figure 4:**
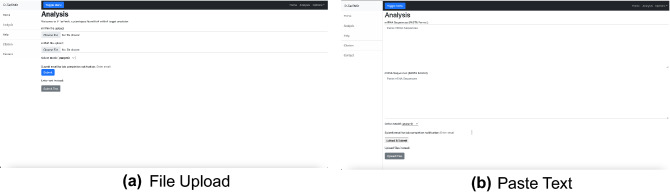
Screenshots of the P-TarPmiR web server job submission page, (**a**) for file upload and (**b**) for direct text input of sequences. Both types of submissions include the ability to add an email so the web server can notify the user when the job is complete. This functionality is particularly useful for large jobs.

A user-friendly web server was developed to perform miRNA target prediction using the P-TarPmiR algorithm. The web server allows users to upload miRNA and target files or copy text into the available text boxes for prediction (Fig. 4). Sequence length limits are imposed to limit the strain on the external remote compute resource. The typical run time for a submission of maximum size is 4 h. The time in the queue is dependent on the job load experienced by the remote compute resource.

**Figure 5:**
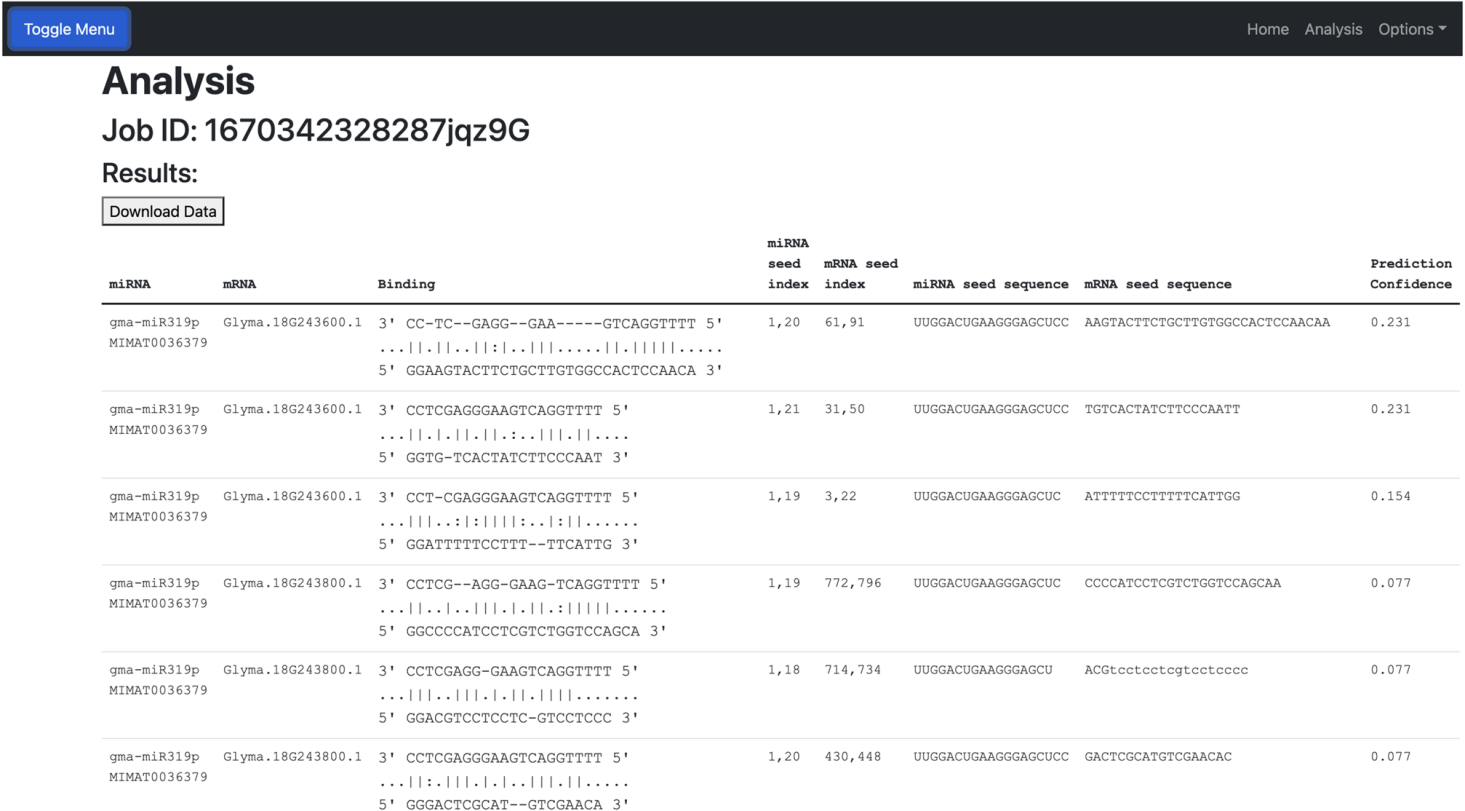
A screenshot of example results of a job submission on P-TarPmiR web server. The results page includes the predicted binding of the seed location, the index of the predicted seed location on the miRNA and mRNA, the miRNA and mRNA seed sequences, and the prediction confidence of the miRNA–mRNA pair.

Once the job is complete, prediction results are displayed and available for download in CSV format. In addition to the target prediction confidence, the results include the target region location and sequence (Fig. 5).

We have also made available the code and installation instructions such that users can install and run PTarPMir locally at https://github.com/GreenCUBIC/PTarPmiR.

### P-TarPmiR applied to soybean genome

P-TarPmiR trained on all plant data described in Table 1 was applied to available soybean miRNA and mRNA. Following fivefold cross-validation across all known soybean interactions, a confidence threshold of 0.98 was selected, which represents a recall and precision of 0.90 ± 0.01 and 1.0 ± 0.00 respectively. This threshold was applied to the target predictions to arrive at a list of high-confidence interactions for each soybean miRNA. GO term enrichment was computed among the high-confidence mRNA targets predicted for each miRNA, using the PANTHER package^31,32^. This resulted in 3 tables of enriched GO terms for each miRNA, one for each annotation set (biological process, molecular function, and cellular component). All target predictions and their relative confidence levels and GO term enrichment results are available at https://doi.org/10.5683/SP3/LOD4E3

## Discussion

We have leveraged a newly released large database of plant-specific miRNA–mRNA interactions to retrain a state-of-the-art predictor to achieve near-perfect performance over four plant test species. We have demonstrated that plant-specific predictors are more effective than cross-kingdom or multi-kingdom classifiers.

In our study, we demonstrated that the inclusion of plant interaction data in the training data resulted in a statistically significant difference in the performance of the classifier. “Human+Plant” and “Plant” classifiers performed significantly better than the two other classifiers that were not trained on a large amount of plant interaction data. Moreover, we demonstrated that the classifier trained strictly on plant interaction data can result in a consistent increase in performance over the “Human+Plant” classifiers even with the reduction in the size of the training dataset.

This study has shown that the inclusion of species-specific data increases the precision of the target predictions while maintaining a high recall. Over four test species, the “Human” classifier was applied to four different plant interaction datasets where the experimental design used here ensured that there was no significant sequence similarity between the training and test sets. Thus resulting in an average recall of 0.996 and average precision of 0.680. The “Human” classifier was able to predict high-confidence miRNA-mRNA interactions with high recall, but the precision was lower than the classifiers trained using plant-based data. These findings are in line with the original TarPmiR manuscript, where Ding et al. reported a significant drop in performance when independent test data were used to evaluate their method^8^. The lower precision observed in this study between the “Human” classifier and the “Human+Plant” classifier could be a measure of the generalizability of the “Human” classifier on a cross-kingdom case. In Ref.^33^, we have previously demonstrated the value of species-specific training sets for miRNA discovery. The present study reinforces this finding but for the case of miRNA target prediction.

The training and test sets exhibited some class imbalance which would affect the precision, accuracy, and AUC scores; however, the recall performance metric would be unaffected by the imbalance. Along with the other metrics, the recall scores of the classifier increased between the animal-based classifier and the plant-based classifier. When the methods are deployed to examine miRNA targets within a complete genome, we expect the class imbalance to be much higher since most miRNA–mRNA pairs will not represent actual interacting pairs.

psRNATarget^12^, a commonly used plant-specific *ab initio* miRNA target predictor reported an average recall of 0.431, an average precision of 1.0, and an average accuracy of 0.732 on our test sets. Relative to psRNATarget, our “Plant only” P-TarPmiR predictor dramatically improved prediction recall (0.993) with minimal reduction in precision (0.984). Similarly, PTarPmiR outperforms TAPIR and Targetfinder in terms of recall. TAPIR and Targetfinder resulted in average recalls of 0.264 and 0.374 and average precision of 0.999 and 1.0 respectively on our test sets.

To explore the ability of our plant-specific PTarPMir model to recover atypical miRNA–mRNA interactions, a subset of miRNA–mRNA interactions was extracted from TarDB to test the performance of psRNATarget and PTarPmiR, specifically on non-canonical interactions. 371 interactions were determined to be atypical since they do not follow the definition of a canonical interaction^34^. Among this subset, psRNATarget resulted in a recall of only 0.108 at the default threshold of an expectation of 3, while PTarPmR resulted in a recall of 0.958 at a similar threshold. The high overall precision and low general recall highlight the fact that ab initio miRNA target predictors can only recover canonical interactions, as discussed by Dai et al.^12^.

## Conclusion

In this paper, we adapted TarPmiR, an animal miRNA target predictor, for use on plants. TarDB, a new plant miRNA–mRNA interaction database, was used to create plant-specific training sets for the miRNA target predictor. We demonstrated that an animal-based target predictor cannot adequately perform on plant data. We determined that a significant amount of plant interaction data could significantly improve the target predictor. Surprisingly, we discovered that a plant-only dataset consistently performed better than the multi-kingdom training sets. P-TarPmiR, the final plant-based miRNA target predictor, is available for use at ptarpmir.cu-bic.ca.

Future work will examine the use of a reciprocal perspective (RP) to improve plant-specific miRNA target predictions. Although RP, a cascaded semi-supervised machine learning method, was first developed to enhance protein–protein interaction prediction, it has shown great promise in other pairwise prediction tasks, including miRNA target prediction in animals^35^.

Future work will also apply P-TarPmiR to soybean to discover miRNA that may play a role in early flowering and resistance to pathogens. miRNA-mediated gene regulation plays an important role in many animal and plant processes. Plants can alter their gene expression in response to stressors^36,37^. However, several studies have indicated that, in addition to intra-species gene regulation, miRNA can be transmitted between species and inhibit another species’ gene expression^38–42^. Inter-species miRNA targeting has been reported in several plant–pathogen relationships where pathogen miRNA target host genes or host miRNA target pathogen genes^43–48^.

In soybean, the differential expression of many miRNAs has been linked to the presence of the soybean cyst nematode (SCN)^37,49^. Additionally, the differential expression of exocyst genes in soybean has been tied to the facilitation or suppression of SCN parasitism in the plant^50^. Soybean is a major legume crop in North America, resulting in billions of dollars of revenue^51^. SCN, a highly specialized plant-parasitic nematode, is a major pathogen of soybean worldwide, causing both significant yield and grain quality losses^51^. While no direct evidence has been identified thus far, the possibility of cross-kingdom interaction between SCN miRNAs and soybean mRNA was recently investigated using a predictor-based approach^52^. Future work will examine animal-based, plant-based, and multi-kingdom classifiers to determine which approach is most useful for cross-kingdom host-pathogen miRNA target prediction. More broadly, PTarPmiR could also be applied to other plant species to further elucidate plant gene regulation as it relates to all kinds of biological processes, such as development, yield maximization, and stress adaptations^36^.

## Methods

### Data retrieval

All the plant miRNA–mRNA interactions listed in the TarDB database^30^ were downloaded. TarDB is a recently released database of high-confidence plant miRNA–mRNA interactions, including binding site information, as determined by miRNA-triggered phasiRNA loci, cross-species conserved targets, and degradome/PARE (Parallel Analysis of RNA Ends)^30^. The miRNA and mRNA sequences of 42,692 interactions could be retrieved. Additionally, 70 of the miRNA and mRNA listed in the *Arabidopsis thaliana* miRNA–mRNA interactions from German et al. were also retrieved^53^. The *A. thaliana* data serves as the source of plant interaction data for the minimally augmented multi-kingdom classifier discussed further in “Discussion” section. Lastly, the miRNA and mRNA of 18,514 of the *Homo sapiens* miRNA–mRNA interactions listed in the Human CLASH dataset originally used to train TarPmiR were also retrieved miRBase and NCBI GenBank^54^. These three sources resulted in the TarDB, *A. thaliana*, and *H. sapiens* positive datasets, respectively.

Negative miRNA–mRNA interaction examples were created using the methodology described in Ding et al. to form the corresponding TarDB, *A. thaliana*, and *H. sapiens* negative sets^8^. In brief, the negative examples were selected by examining potential negative sites on an mRNA from a documented positive interaction that did not overlap with the positive site and had a similar CG dinucleotide frequency to the positive site. For each mRNA, the negative site exhibiting the lowest folding energy was used as the final negative exemplar^8^.

### Feature extraction

The TarPmiR^8^ software package was modified to extract 11 features from the miRNA and target site pairs, including folding energy, seed match, accessibility, AU content, m/e motif, the total number of paired positions, length of the target mRNA region, length of the largest consecutive pairings, the position of the largest consecutive pairings relative to the 5$^{\prime }$ end of the miRNA, the number of paired positions at the miRNA 3$^{\prime }$ end and the difference between the number of paired positions in the seed region and in the miRNA 3$^{\prime }$ end. TarPmiR utilizes the miRanda software^9^ to find and extract features from seed regions on the miRNA and mRNA sequences.

### Training set development and experimental set-up

For each test species, the following steps were used to create the training sets and the test sets, such that none of the training data shared significant sequence identity with any of the evaluation data from the test species. The plant training sets were used to train one of the four classifiers during the four experiments. The test set was used to determine the performance of each classifier. For each plant test species, the training set contained all examples from the TarDB dataset excluding those involving miRNA exhibiting greater than 70% sequence identity with any miRNA from the test species. All TarDB examples from the target organism formed the test set. Examples with miRNA sharing sequence identity with any examples from the *A. thaliana* dataset miRNA were also excluded from the test set. Test examples with duplicate features were also removed.

All classifiers used Random Forest models with 13 trees (from Ding et al.). The first experiment replicated the original TarPmiR classifier where the Random Forest classifier was trained on the Human CLASH positive and negative data. The second experiment consisted of training the model on a training set including the *H. sapiens* and the *A. thaliana* positive and negative sets. A third experiment trained the model on the *H. sapiens* dataset along with the plant positive and negative datasets for that experiment. The training sets in experiments two and three contain different proportions of plant interaction data. This will test how much plant data is required to perform well. In the fourth experiment, the model was trained on only the plant training sets in each experiment.

### Web server

The miRNA Target prediction web server was developed using the Node.js Express framework. Common JavaScript libraries were used to develop a user-friendly interface. The web server runs with the support of Digital Research Alliance of Canada, a remote cloud-based compute resource that allows the submission of multiple concurrent jobs. The web server was containerized using Docker to ensure the portability and scalability of the web server. The web server is freely available for use at ptarpmir.cu-bic.ca

### P-TarPmiR applied to soybean

A model trained on the entirety of the TarDB database was applied to all 756 mature miRNA available for soybean (*Glycine max*) in miRBase^55^ and all 88647 transcripts available in version Wm82.a2 of soybean from Soybase^56^. The threshold to determine high-confidence interactions was determined using fivefold cross-validation of the soybean interactions extracted above. GO term enrichment analysis was applied to all high-confidence interactions predicted for each mature miRNA using PANTHER^31,32^.

## Data Availability

All sequence and interaction data used to train and test the methods are available from public repositories (see details in the manuscript).
